# What the future holds for Biomedical Engineering Online?

**DOI:** 10.1186/s12938-019-0702-x

**Published:** 2019-07-24

**Authors:** Ervin Sejdić, Fong-Chin Su

**Affiliations:** 1 Department of Electrical and Computer Engineering, Swanson School of Engineering, University of Pittsburgh, Pittsburgh, PA 15261 USA; 2 Department of Bioengineering, Swanson School of Engineering, University of Pittsburgh, Pittsburgh, PA 15261 USA; 3 Department of Biomedical Informatics, School of Medicine, University of Pittsburgh, Pittsburgh, PA 15261 USA; 4 Intelligent Systems Program, School of Computing and Information, University of Pittsburgh, Pittsburgh, PA 15261 USA; 5 Department of Biomedical Engineering, Medical Device Innovation Center, National Cheng Kung University, Tainan, 701 Taiwan, ROC

## Abstract

The future of biomedical engineering is exciting, and prospects for biomedical engineering journals are rapidly growing. In this editorial, a brief history of *Biomedical Engineering Online* is outlined, along with our plans for future directions of the journal.

## Introduction

We live in exciting times for biomedical engineering. The rapid growth of various technical fields such as artificial intelligence, material science, data science, fluid dynamics, continuum mechanics, energy and all other fields are rapidly changing the face of biomedical engineering. What was unimaginable 5–10 years ago, it’s a common practice nowadays. Just consider how the development of graphical processing units and their lowering costs have enabled researchers across the globe to utilize various machine learning techniques to analyze tens of thousands of radiological images in a quick manner and publish those results within the months of the inception of their research idea. Such a rapid scientific turnover was not even imaginable a few years ago. These developments are not only changing the content of most scientific journals, but also the quality of published manuscripts. Most journals are receiving manuscripts of a higher quality, and many papers are rejected at the editorial level, which otherwise would have been published 5–10 years ago. Hence, *Biomedical Engineering Online* is under the same pressure to attract higher quality contributions.

## The past of the journal

*Biomedical Engineering Online* first launched as an open access journal published by BioMed Central in 2002. While initially there has been some resistance by researchers to pay for open access fees, as some researchers argued that only authors of a low-quality manuscript are paying for their manuscripts to be published. Fast forward to 2019, it is almost a standard procedure these days, and no one is questioning the quality of open access journals anymore, as some of the most prestigious journals are open-access journals (e.g. *Nature Communications*). Furthermore, several business changes have also occurred that had an impact on the journal. In October 2008, BioMed Central was acquired by Springer Science + Business Media. In 2015, Springer Nature was formed by a merger of several publishing groups such as Springer Science + Business Media, Nature Publishing Group, Palgrave Macmillan, and Macmillan Education. While these mergers did not have an immediate impact on the journal, the latest merger, that is, the forming of Springer Nature, will have a major impact on our future as it will be outlined in the next section.

The inaugural Editor-in-Chief was Dr. Alvin Wald from Columbia University, USA. Professor Kenneth Foster from the University of Pennsylvania (USA) took over in 2006, with Prof. Fong-Chin Su from National Cheng Kung University in Taiwan joining as a co-editor-in-chief in 2014.

The journal received its first impact factor in 2008, and it was 1.800. Over the years, the Impact Factor maintained a healthy status ranging from 1.119 in 2010 to 2.013 in the most recent 2018 Journal Citation Report. Based on these Impact Factors, the journal maintained its position in the second and third quartiles of Impact Factors within the biomedical engineering group of journals. According to the latest numbers provided by Clarivate Analytics, the journal is ranked 52nd out of 80 biomedical engineering journals. Professors Foster and Su maintained these great rankings all these years by attracting excellent manuscripts, not an easy job. They also formed a great team of Associate Editors, who work hard to obtain necessary high-quality peer reviews for a growing number of submissions. It should be noted that the journal has established a fairly low acceptance ratio, and it typically hovers around 20–30% over the past several years.

After many years of tirelessly supporting the journal and the field, Professor Foster has decided to retire from the position of an Editor-in-Chief. We sincerely thank him for all his hard work and dedication, and we are happy to see him remain as a member of the editorial board, especially during the transition period ahead of us.

## The future of the journal

Scientific publishing is a challenging business due to multiple stakeholders, but they all seem to have the same desire. On one hand, it’s driven by publishing houses, who are mostly for-profit organizations that desire to publish quality journals that will have value to the research communities they serve and attract readers to these journals (we are referring to reputable publishing houses here). On the other hand, scientists and researchers also desire to publish in high impact journals, as in today’s scientific surroundings, it typically attracts more research funds, translation of knowledge and career promotions to these researchers. It should be acknowledged that the Impact Factor alone, is an imperfect measure of research quality. The publisher (BioMed Central) signed up to the San Francisco Declaration on Research Assessment (DORA) in 2017 [1] and is committed to moving away from this metric as a single measure of quality. Additional metrics such as Source normalized impact per Paper (SNIP) and SCImago Journal Rank (SJR), that incorporate citation data from a larger database of journals should also be considered. On an individual article level, access data and measures of online visibility, such as Altmetric scores, also indicate the value to readers.

In light of the rapid advance of technology in the field, we have decided to challenge ourselves, and come up with a bold vision that we will try to achieve over the next few years.

And, drumroll please! Our bold vision is to focus on the value of our content to the wider community and become a biomedical engineering journal that is well known for publishing high quality research. We will endeavor to publish more articles that showcase novel advances this growing field. In the medium term, we aim to be ranked in the first quartile of Impact Factors, within the biomedical engineering category of the Journal Citation Reports.

In order to achieve this vision, we will definitely need to alter our approach in attracting and selecting work that reflects the rapid development of the field. What it all means? It does not necessarily mean that we will cut the acceptance ratio by half, as the current acceptance ratio of 20% is typical of other prestigious biomedical journals. However, it does signify that we will be seeking manuscripts that have broader and/or stronger impacts on the field. In other words, we will probably not consider manuscripts dealing only with healthy controls anymore, as those manuscripts typically represent early stage results that are a long shot away from a potential long-term impact. Of course, the word probably is emphasized here, as some manuscripts may represent the analysis of data from several hundreds of healthy controls, which may be of interest to a broader audience. Also, manuscripts covering machine learning algorithms which are developed and tested based on well-known open datasets will generally not be of our interest in future. Our experiences have shown that these manuscripts have allow utility to others, as these algorithms are typically not generalizable to broader and more diverse datasets.

To achieve our new vision, we will also implement several procedural changes. First, it deals with special issues. From now on, we will require that each proposal for a special issue has at least three guest editors from different geographic locations in order to prevent all manuscripts representing uniform opinions. This will be requested for all special issues unless the special issue is from selected papers of an international conference/symposium. Similarly, these proposals will also need to outline the special issues in detail and list the names of potential contributors and titles of their potential manuscripts. While we understand that it is impossible to predict all contributions, we would ask guest editors to have at least 5–10 confirmed manuscripts from a diverse set of authors before the approval of the special issue. Second, we would like to welcome more review articles in the journal. However, we sincerely encourage the authors to work with the editor-in-chief to develop an appropriate topic and potentially an outline of the review paper before submitting the full draft for consideration. This process will ensure that the manuscript is of interest to the journal, and that it covers necessary points. Third, we have managed to include editorial edits for accepted manuscripts within the article-processing charge, and we will pay even more attention to the writing styles presented in submitted manuscripts. Poorly written manuscripts, even those representing innovative ideas, may be triaged at the editorial level. Some authors will be encouraged to seek help with writing, before re-submitting their work consideration for review Authors of rejected manuscripts, that are otherwise scientifically sound, will also be given the opportunity to transfer their submission to an alternative journal at the publisher, saving time and helping to reduce the burden on peer-reviewers.

Our focus on quality may take 2–3 years to have an impact on journal metrics. To improve the value of the journal in the short term, we will also be working closely with our expert panel of Associate Editors to reduce our editorial triage and peer-review times at the journal. We would like to offer the best possible service to the community, with a rapid route to publication and all the benefits of visibility that open access brings.

## Concluding remarks

*Biomedical Engineering Online* has become one of the staple biomedical engineering journals due to the leadership of our previous Editor-in-Chiefs and all editorial board members. It is the time for the journal to grow even more, and we outlined a bold vision that we want to achieve over the next several years. As such, we encourage you to submit your most impactful and most novel work to the journal, and we will work with your team to publish it in the journal.
